# Effects of microvirin monomers and oligomers on hepatitis C virus

**DOI:** 10.1042/BSR20170015

**Published:** 2017-06-30

**Authors:** Yuan-Qin Min, Xu-Chu Duan, Yi-Dan Zhou, Anna Kulinich, Wang Meng, Zhi-Peng Cai, Hong-Yu Ma, Li Liu, Xiao-Lian Zhang, Josef Voglmeir

**Affiliations:** 1 State Key Laboratory of Virology, Department of Immunology, Hubei Province Key Laboratory of Allergy and Immunology, School of Medicine, Wuhan University, Wuhan, People’s Republic of China; 2 Glycomics and Glycan Bioengineering Research Center (GGBRC), College of Food Science and Technology, Nanjing Agricultural University, Nanjing, People’s Republic of China; 3 Department of Microbiology, School of Molecular and Cellular Biology, University of Illinois at Urbana-Champaign, IL 61801, U.S.A.; 4 Department of Plant Pathology, Nanjing Agricultural University, Nanjing, People’s Republic of China

**Keywords:** high-mannose glycan, hepatitis C virus, lectin oligomer, Microvirin

## Abstract

Microvirin (MVN) is a carbohydrate-binding protein which shows high specificity for high-mannose type N-glycan structures. In the present study, we tried to identify whether MVN could bind to high-mannose containing hepatitis C virus (HCV) envelope glycoproteins, which are heavily decorated high-mannose glycans. In addition, recombinantly expressed MVN oligomers in di-, tri- and tetrameric form were evaluated for their viral inhibition. MVN oligomers bound more efficiently to HCV virions, and displayed in comparison with the MVN monomer a higher neutralization potency against HCV infection. The antiviral effect was furthermore affected by the peptide linker sequence connecting the MVN monomers. The results indicate that MVN oligomers such as trimers and tetramers may be used as future neutralization agents against HCV infections.

## Introduction

Hepatitis C virus (HCV) is a major health concern with estimated 130–200 million people infected worldwide [1]. Patients developing a chronically persistent infection have a high risk of cirrhosis and hepatocellular carcinoma [2]. So far, no vaccination for any of the seven HCV genotypes (type 1–7) is available [3–5]. Current treatments for hepatitis C include PEGylated interferon-α or ribavirin-based therapies [6,7]; however, responses to these treatments vary significantly with viral genotype and host genetic background [8]. Recently, treatments involving drugs such as telaprevir and boceprevir, which directly inhibit viral HCV NS3/4A protease [9,10], have been considered a breakthrough therapy for the specific treatment of HCV genotype 1b infection by the FDA in 2011 [11]. However, these protease inhibitors are also less effective against genotype 3 in all seven HCV genotypes, and drug resistance may develop due to rapid mutational adaption [12]. More recently, the discovery of the nucleotide analogue sofosbuvir, which is capable of inhibiting the NS5B polymerase in HCV with high efficacy, gives hope of targeting HCV infections [13]. However, the safety of this compound still needs to be evaluated in clinical trials. In addition that most of these therapies are rather cost intensive and have possible side effects [12–15], and there is the risk that viral strains resistant to these treatments will develop. Therefore, the discovery of novel compounds with antiviral mechanisms distinct from those of known anti-HCV agents, and which can be applied alone or in a combination with existing drugs, is highly desirable and much needed.

HCV is an enveloped virus with two highly glycosylated proteins, E1 and E2, which mediate the entry of HCV into hepatocytes. In comparison with the high sequence variability occurring in the viral genome, most glycan sites on E1 and E2 are highly conserved across the seven genotypes [16], indicating their critical role in the virus life cycle. There are five and eleven N-linked glycosylation sites reported on E1 and E2 respectively. Analysis of the N-glycans of the E2 protein expressed in recombinant form showed that all N-glycosylation sites are decorated with high-mannose ype N-glycans except for the Asn^423^ or Asn^430^ N-glycosylation sites, which may also be decorated with complex type N-glycans [17]. Different N-glycosylation sites on the E1 or E2 proteins play different roles in HCV life cycle [18,19]; mutational studies suggest that the Asn^423^ or Asp^448^ of the E2 glycoprotein are critical for virus entry and their modification results in loss of viral infectivity [19]. Furthermore, N-glycosylation may be essential for assembling the E1/E2 heterodimer complex which is crucial for effective binding to the cellular entry receptors and membrane fusion with the host cell [20]. Based on the importance of the glycosylation for the viral activity, we presumed that lectin binding might impair proper E1/ E2 complex formation and therefore might hamper HCV virulence.

**Figure 1:**
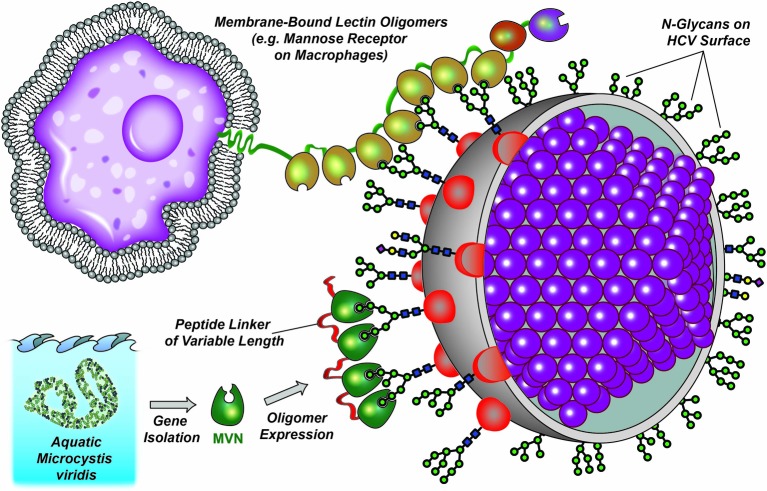
Natural and artificial oligomerization of lectins The pearl chain-like organization of naturally occurring lectin oligomers (top) and artificially oligomerized MVN mimics (bottom).

Several studies have indicated that lectins can block virus infection by binding to the envelope glycoproteins. For example, *Hippeastrum hybrid* agglutinin [21], *Musa acuminate* BanLec [22], griffithsin lectin (GRFT) from a red algae [23], and the cyanobacterial lectins cyanovirin-N (CV-N) [24] and microvirin (MVN) [25] have been shown to inhibit HIV infection via binding to the envelope glycoprotein gp120. More recently, the effects of lectins on HCV infection were also tested with similar results: both GRFT and scytovirin from blue algae have been shown to inhibit HCV at picomolar and nanomolar concentrations [26,27]. CV-N, *Galanthus nivalis* agglutinin (GNA) and *Microcystis viridis* lectin were also reported to block HCV infection while in different virus entry stages through recognition of different glycan structures [28–30]. Therefore, lectins show great potential for use in synergistic therapies treating viral infections. In the present study, we utilized HCV as a target to evaluate the antiviral potency of MVN and the recombinantly engineered MVN oligomers (Figure 1). This pearl chain-like arrangement is similar to the organization of C-type lectins, such as the mannose receptor CD206 which is involved in viral recognition on macrophage cell membranes [31].

## Materials and methods

### Materials

Huh7.5.1 cells were cultured in Dulbecco’s modified Eagle’s medium (DMEM; Hyclone) supplemented with 10% fetal bovine serum (FBS; Gibco) at 37°C in a 5% CO_2_ atmosphere. Cell culture-derived HCV (HCVcc, genotype 2a, JFH1 isolate) expansion was performed using Huh7.5.1 cells in a safety level 2 laboratory. Horseradish peroxidase (HRP)-conjugated anti-His monoclonal antibody (mAb) was purchased from ProteinTech (Wuhan, China); *Galanthus nivalis* lectin (GNA) was purchased from Sigma–Aldrich (St. Louis, MO, U.S.A.); the gene sequence encoding the monomeric MVN lectin was synthesized by ZoonBio (Nanjing, China). Oligonucleotide primers were synthesized by GenScript (Nanjing, China). DNA polymerases were purchased from Takara (Dalian, China); *Escherichia coli* competent cells, restriction endonucleases and T4 ligases were obtained from Thermo Fisher Scientific (Shanghai, China); DNA Gel Purification and Plasmid Extraction kits were from Axygen (Beijing, China). All chemicals used in the present study were of analytical grade or higher.

### Construction of expression plasmids

The pET-30a-MVN (monomer) vector was constructed by inserting the MVN coding sequence into a pET-30a vector using NdeI and XhoI restriction sites. pET-30a-MVN dimer 2 (2S), MVN dimer 6 (2M) and MVN dimer 10 (2L) vectors were constructed by inserting two MVN coding sequences sequentially into the pET-30a vector using NdeI*/Bam*HI and BamHI/XhoI restriction sites. The pET-30a-MVN trimer 2 (3S), MVN trimer 6 (3M) and MVN trimer 10 (3L) vectors were constructed by inserting three MVN coding sequences sequentially into pET-30a vector using NdeI/ BamHI, BamHI/EcoRI and EcoRI/XhoI restriction sites. The pET30a-MVN tetramer 2 (4S), MVN tetramer 6 (4M) and MVN tetramer 10 (4L) vectors were constructed by inserting four MVN coding sequences sequentially into pET-30a vector using NdeI/BamHI, BamHI/EcoRI, EcoRI/SacI and SacI/XhoI restriction sites. Spacer peptides between the MVN monomer units with 6 or 10 amino acids of lengths were introduced using DNA-overhangs with 6 or 12 additional base pairs between the priming sequence and the restriction site overhang on the 5′ end (Supplementary Table S1). All MVN coding sequences were obtained by PCR amplification using Primestar DNA polymerase. PCR reactions were performed as recommended by manufacturer, with annealing temperatures set at 55°C. After restriction, enzymatic digestion and ligation, recombinant plasmids were transformed into *E. coli* Top 10 competent cells. Candidate clones were selected by colony PCR and confirmed to contain the expected plasmid constructs by Sanger DNA sequencing (Genscript, Nanjing).

### Expression and purification of the recombinant MVN oligomers

Plasmids containing different MVN variants were transformed into *E. coli* BL21 (DE3) competent cells, and transformants were grown in 400 ml of fresh LB medium under continuous shaking (250 rpm) at 37°C until OD_600_ values of cells reached 0.5–0.6. To induce the expression of recombinant proteins, isopropyl β-D-thiogalactopyranoside (IPTG) at a final concentration of 1 mM was added to the cell culture. After 3 h of continuous shaking at 25°C, cells were harvested by centrifugation at 5000 ***g*** for 15 min, resuspended in a lysis buffer (50 mM Tris, 100 mM NaCl, 1% Triton X-100, pH 8.0) and disintegrated by sonication (40 on/off cycles with 20 μm amplitude for 15 s at 4°C). The resulting cell lysates were centrifuged at 20000 ***g*** for 30 min and the supernatants were loaded onto Ni^2+^-nitrilotriacetate affinity chromatography columns (Ni-NTA) (Qiagen, 2 ml bed volume) equilibrated with washing buffer (50 mM Tris, 50 mM NaCl, pH 8.0). C-terminally His-tagged proteins were eluted with elution buffer (50 mM Tris, 50 mM NaCl, 300 mM imidazole, pH 8.0). The fractions with the highest absorbance at 280 nm (approximately 20–50% of the total protein retained, Supplementary Table S2) were combined and desalted with PD-10 protein purification columns (GE Healthcare, pre-equilibrated with 20 mM piperazine) using 20 mM piperazine as mobile phase. The desalted proteins were further purified using prepacked Q Sepharose XL columns (GE Healthcare, 1 ml bed volume) by applying a linear gradient of NaCl for elution (from 0 to 1 M NaCl in 10 min, Supplementary Figures S1–S10). The process of purification was further evaluated by SDS/polyacrylamide gel electrophoresis (SDS/PAGE) and visualized by Coomassie Brilliant Blue G-250 staining. Samples aliquots were stored at −80°C until further usage.

### N-glycan analysis of hepatitis C virus particles

Crude isolates of HCV were used for the preparation of N-glycans after pepsin digestion and PNGase F treatment as previously described [32]. The released N-glycans were fluorescently labelled with 2-aminobenzamide (2-AB) based on the method described by Bigge et al. [33] and further analysed by hydrophilic interaction liquid chromatography (HILIC) ultra performance liquid chromatography (UPLC) using the same conditions as described by Du et al. [34]. N-glycan structures were annotated using the GlycoBase N-glycan repository [35] and UPLC fractions of the dominant peaks were manually collected. After solvent evaporation using vacuum centrifugation, samples were subjected to mass spectrometric analysis and interpreted using GlycoWorkbench version 1.1. [36].

### Estimation of MVN binding efficiency by double-lectin sandwich ELISA

GNA, serving as scavenging agent, was used to coat 96-well EIA plate (incubation in 5 μg/ml in phosphate buffered saline (PBS) for 60 min at 37°C). Unbound GNA was further removed from the wells by washing with the PBS buffer. Copies of HCVcc or serum (2 × 10^5^) from HCV (gp1) positive patients (100 μl, undiluted or 6-fold diluted) were added and incubated for 1 h at 37°C. Unbound HCV was removed by washing with PBS. The recombinant His-tagged MVN mono- and oligomers (final concentration of 0.4 and 4 μg/ml) were added to the captured HCV virions and incubated at 37°C for 60 min. Binding was visualized using HRP-conjugated anti-His mAB and 3,3′,5,5′-tetramethylbenzidine (TMB) as a chromogenic reagent followed by quenching the reaction with 100 μl of 2 M H_2_SO_4_. The further quantification was carried out on a microplate reader (PerkinElmer Victor X5) at an absorbance wavelength of 450 nm. EIA samples plates lacking GNA coating were used as negative controls.

### Neutralization assays of HCV by MVN oligomers

Three multiplicities of infection (MOI) of HCVcc in 400 μl were incubated with different concentrations of MVN for 1 h at 37°C. The mixture was then added into Huh7.5.1 cells (1 × 10^5^ copies/well) seeded on 24-well plates. After 6 h incubation at 37°C, the unbound HCVcc was removed by washing with PBS buffer, before the addition of fresh complete medium to the wells. Cells were further collected for HCV inhibition assays after 72 h. GNA-treated samples were used as a positive control. Culture medium and Huh7.5.1 cells without HCVcc were used as negative controls.

### Quantitative real-time PCR

Total cellular and viral RNA were isolated using phenol/chloroform extraction and reverse transcribed using a random primer with the ReverTra Ace Alpha kit (Toyobo, Dalian). Real-time PCR quantification was performed using the SYBR Green Real-time PCR Master Mix (Plus) kit (Toyobo, Dalian). Relative HCV genome RNA levels were calculated by the 2^−ΔΔ*C*^_T_ method with GAPDH mRNA as an internal control and were shown as relative change by normalizing to the untreated control samples (Primer pairs RT-HCV and RT-GAPDH respectively, Supplementary Table S1).

## Results

### Construction, cloning, expression and purification of recombinant MVN oligomers

**Figure 2:**
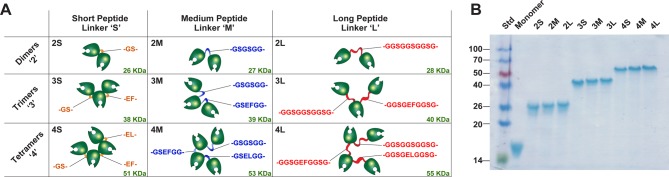
Overview of generated MVN oligomers (**A**) schematic representation of the linked MVN monomeric units and designation of MVN oligomers; (**B**) SDS/PAGE analysis of purified proteins.

MVN dimers, trimers and tetramers were generated to elucidate the oligomerization effect on lectin binding and antiviral activity. Recombinant MVN variants contained tandem repeats of MVN, with C-terminus of one subunit connected via 2 to 10 amino acids to the N-terminus of the next subunit (Figure 2A). The MVN oligomers contained an N-terminal hexahistidine tag to facilitate the protein purification. The purity of the preparations was evaluated by SDS/PAGE (Figure 2B). The major bands representing the MVN variants could be clearly observed. The sequences of the short, medium and long oligopeptide linkers used for the generation of the recombinant MVN oligomers are shown in Figure 2(A).

### N-glycan profile of hepatitis C virus particles

The N-glycan profile of the HCV particles was analysed by UPLC and MALDI-TOF-MS/MS, and was found to consist of complex and hybrid N-glycans with terminal galactose, and partly decorated with core fucose (Supplementary Table S3, Supplementary Figures S11–S17). In addition, high-mannose type N-glycans with 5 and 6 mannose units were also found.

### MVN oligomer binding to hepatitis C virus

**Figure 3:**
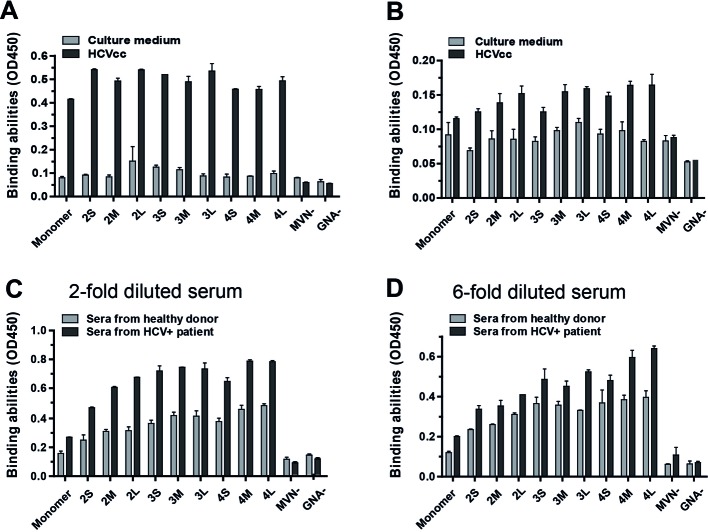
Assessment of the MVN mono- and oligomer binding to HCV virions by double-lectin sandwich ELISA (**A** and **B**) detection of HCVcc (gp2a) with MVN and its oligomers at concentration of 4 and 0.4 μg/ml respectively; (**C** and **D**) detection of HCV from HCV patient sera (gp1) diluted 1 and 6 times respectively.

In order to test the effect of MVN and its oligomers on HCV, a double-lectin sandwich enzyme-linked immunosorbent assay (ELISA) assay was performed. While the use of MVN oligomers at a concentration of 0.4 µg/ml shows moderate binding to the virus, the binding is significantly enhanced when the oligomer concentration were increased to 4 µg/ml. However, the degree of oligomerization did not affect binding activity significantly, as shown with HCVcc (Figure 3A). In addition, an improved ability to bind to HCVcc was observed with increased oligopeptide linker length (Figure 3B). Furthermore, the binding of MVN oligomers to HCV particles derived from patient sera was investigated (Figure 3C and D). Although the background signal in sera from healthy donors was rather high, which might be caused by unspecific binding to other mannose-containing glycans, HCV binding signals for both healthy and patient donors also increased with the degree of MVN oligomerization and the peptide linker length.

### Neutralization of hepatitis C virus by MVN oligomers

**Figure 4:**
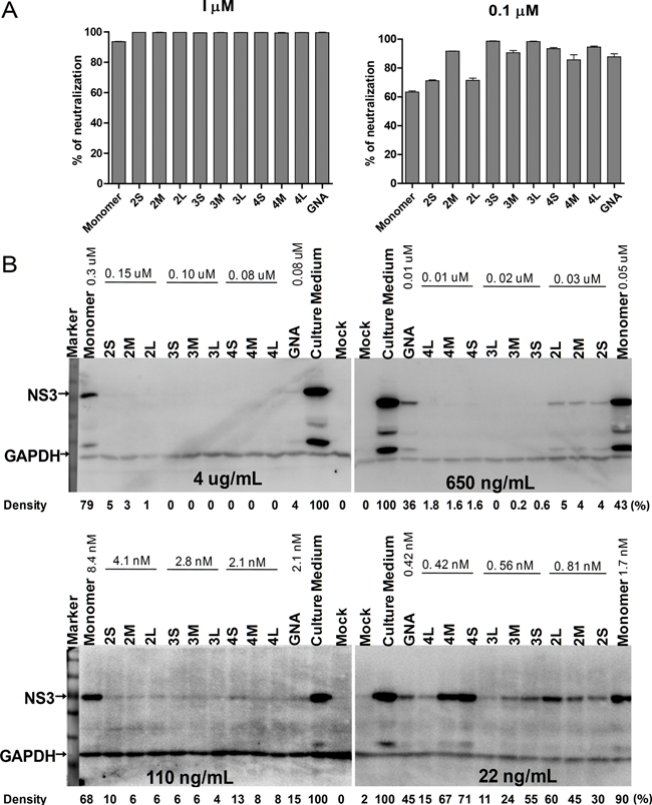
HCV infection neutralization (**A**) HCV RNA levels measured by RT-qPCR; (**B**) HCV NS3 protein detected by Western blot. The NS3 protein levels are normalized to GAPDH by grey scanning analysis using the ImageJ software, and the density results are shown as percentage of the culture medium-treated group.

The ability of MVN mono- and oligomers of neutralizing the HCV was evaluated using an HCVcc-Huh7.5.1 infection system by measuring HCV RNA expression levels with RT-qPCR. In addition, the presence of the virus was also monitored by Western blot detection of the virus protease NS3. We found that all MVN oligomers can completely inhibit HCVcc at a concentration of 1 μM; however, a similar effect was observed for the MVN monomer, which displayed approximately 94% inhibition (Figure 4A). As shown in Figure 4(B), the MVN monomer had even at higher concentrations (4 μg/ml) only limited abilities to neutralize HCVcc. In comparison, the neutralization activities of the MVN dimers and trimmers were significantly better. MVN tetramers also displayed good neutralizing activities against HCV, although it must be noted that the shorter linker forms (4S and 4M) showed lower neutralization than compared with the trimers (especially at low concentrations). This may be caused by a loss in protein stability of the MVN tetramers. In sum, MVN oligomer 3L appeared to possess the highest neutralization activity against HCV, followed by oligomer 4L. Interestingly, apart from the samples tested with 22 ng/ml of MVN variants, most MVN oligomers at higher concentrations (110, 650 and 4 μg/ml) appeared to be more efficient in HCVcc neutralization than GNA, which has previously been described as a potent inhibitor of HCV infection [29].

## Discussion

Lectins are emerging as promising antiviral agents and an alternative to neutralizing antibodies. The selection of efficient monoclonal antibodies is tedious and time consuming due to the weak immunogenicity of viral envelope glycoproteins. In addition, some antibodies, despite binding to the viral proteins, do not have neutralization activity and may interfere with functional neutralization antibodies [37]. There is also a limit on how effectively monoclonal antibodies can target viral epitopes due to their masking with N-glycans [38]. In contrast, lectins and especially lectin oligomers, can target several glycan moieties at once, making it difficult for the virus to adapt rapidly. Engineered lectins with improved properties have been proved to possess potent antiviral activity [39] and low mitogenicity [40]. Proteins such as CV-N have demonstrated that oligomeric lectin variants may be used to effectively neutralize particular envelope viruses such as the human immunodeficiency virus (HIV), influenza and vaccinia viruses [41].

Here, we present engineered oligomers of MVN, a recently isolated lectin from the freshwater cyanobacterium *Microcystis aerugenosa*, which is highly specific for high-mannose structures with α1,2-linkages [42]. Monomeric MVN has been shown previously to neutralize a broad spectrum of HIV-1 strains and, in addition, it was shown to be less cytotoxic than CV-N [25]. By increasing the number of binding units of the lectin and the varying the length of the oligopeptide linkers joining them, we attempted to modulate the neutralization activity of MVN. We found that all MVN oligomers showed improved neutralization of cultured hepatitis C virions and of HCV in human serum samples compared with the MVN monomer. In addition, enhanced neutralization activity was achieved compared with the tested monomeric MVN and GNA lectins, the antiviral potencies of which have been reported previously [43]. Interestingly, MVN tri- and tetramers appeared to have higher anti-HCV activity in comparison with MVN monomers and dimers. Almost complete HCV neutralization was observed at 650 ng/ml as judged by NS3 protease expression. Similar results were reported for recombinant CV-N variants: oligomers were more potent neutralizers of HIV and influenza viruses than the monomer variant; however, CV-N dimers had greater potency in the neutralization of influenza strain x31 (H3N2) than trimers and tetramers [40]. Furthermore, Keeff et al. [41] reported increased stability under physiological conditions of CV-N after oligomerization. We found that longer peptide linkers between MVN oligomers lead to an increased anti-HCV activity, which has also been found to be the case for CV-N. According to Mayo et al. [39], this finding can be explained by the increased steric freedom of the lectin subunits allowing the monomers a better access to mannose residues on the virus surface.

## Conclusion

A series of engineered MVN oligomers with modulated binding and neutralization properties towards the HCV are reported in the present study. By increasing the degree of oligomerization and the length of the oligopeptide linker, neutralization of HCV could be achieved at lower lectin concentrations. The results obtained may have particular significance in light of the applications of MVN in lectin affinity plasmapheresis for dialysis patients, and as an alternative antiviral agent to GNA, a promising agent for the treatment of HCV [44].

## Supporting information

**Supplementary Figure 1:** FPLC chromatogram of the Q Sepharos XL ion exchange purification of the MVN monomer. Fraction 11 and 12 (red arrow) were collected and used for further experiments.

**Supplementary Figure 2:** FPLC chromatogram of the Q Sepharos XL ion exchange purification of the MVN dimer 2S. Fraction 14 and 15 (red arrow) were collected and used for further experiments.

**Supplementary Figure 3:** FPLC chromatogram of the Q Sepharos XL ion exchange purification of the MVN dimer 2M. Fraction 15 and 16 (red arrow) were collected and used for further experiments.

**Supplementary Figure 4:** FPLC chromatogram of the Q Sepharos XL ion exchange purification of the MVN dimer 2M. Fraction 11 and 12 (red arrow) were collected and used for further experiments.

**Supplementary Figure 5:** FPLC chromatogram of the Q Sepharos XL ion exchange purification of the MVN trimer 3S. Fraction 12 and 13 (red arrow) were collected and used for further experiments.

**Supplementary Figure 6:** FPLC chromatogram of the Q Sepharos XL ion exchange purification of the MVN trimer 3M. Fraction 16 and 17 (red arrow) were collected and used for further experiments.

**Supplementary Figure 7:** FPLC chromatogram of the Q Sepharos XL ion exchange purification of the MVN trimer 3L. Fraction 15 and 16 (red arrow) were collected and used for further experiments.

**Supplementary Figure 8:** FPLC chromatogram of the Q Sepharos XL ion exchange purification of the MVN tetramer 4S. Fraction 21 and 22 (red arrow) were collected and used for further experiments.

**Supplementary Figure 9:** FPLC chromatogram of the Q Sepharos XL ion exchange purification of the MVN tetramer 4M. Fraction 15 and 16 (red arrow) were collected and used for further experiments.

**Supplementary Figure 10:** FPLC chromatogram of the Q Sepharos XL ion exchange purification of the MVN tetramer 4L. Fraction 12 and 13 (red arrow) were collected and used for further experiments.

**Supplementary Figure 11:** UPLC chromatogram of HCV-derived N-glycans.

**Supplementary Figure 12:** Mass spectrometric analysis of UPLC elution peak at 24.3 min.

**Supplementary Figure 13:** Mass spectrometric analysis of UPLC elution peak at 27.6 min.

**Supplementary Figure 14:** Mass spectrometric analysis of UPLC elution peak at 28.1 min.

**Supplementary Figure 15:** Mass spectrometric analysis of UPLC elution peak at 29.5 min.

**Supplementary Figure 16:** Mass spectrometric analysis of UPLC elution peak at 31.9 min.

**Supplementary Figure 17:** Mass spectrometric analysis of UPLC elution peak at 32.2 min.

**Supplementary Table 1:** Primers used for cloning MVN and its oligomers

**Supplementary Table 2:** Absorbance values of the elution fractions with the highest protein contents using Ni-NTA purification.

**Supplementary Table 3:** HCV N-glycan composition and possible isomers

## Abbreviations

2-AB: 2-aminobenzamide
CV-N: cyanovirin-N
ELISA: enzyme-linked immunosorbent assay
GNA: *Galanthus nivalis* agglutinin
HCV: hepatitis C virus
HCVcc: cell culture-derived HCV
HILIC: hydrophilic interaction liquid chromatography
HRP: horseradish peroxidase
IPTG: isopropyl β-D-thiogalactopyranoside
mAB: monoclonal antibody
MOI: multiplicity of infection
MVN: microvirin
Ni-NTA: Ni^2+^-nitrilotriacetate affinity chromatography column
PBS: phosphate buffered saline
qRT-PCR: quantitative real-time polymerase chain reaction
SDS/PAGE: sodium dodecyl sulphate-polyacrylamide gel electrophoresis
TMB: 3,3',5,5'-tetramethylbenzidine
UPLC: ultra performance liquid chromatography
