# Supplementary material for: Effects of microvirin monomers and oligomers on hepatitis C virus
Source: Biosci Rep. 2017 Jun 30;37(3):BSR20170015. doi: 10.1042/BSR20170015 (PMC6434159; doi:10.1042/BSR20170015)
Supplement: Supplementary file 1 [file bsr-37-bsr20170015_Supp1.pdf]

Supplementary Information

Effects of Microvirin Mono- and Oligomers on Hepatitis C Virus

Yuan-Qin Min, Xu-Chu Duan, Yi-Dan Zhou, Anna Kulinich, Zhi-Peng Cai, Hong-Yu Ma, Li Liu, Xiao-Lian Zhang and Josef Voglmeir

Supplementary Table 1 Primers used for cloning MVN and its oligomers

Primer names	Sequences (5' - 3')
**MVN-NdeI For**	GGAATTCCATATGCCGAACTTCTCTCACACCTG
**MVN-XhoI Rev**	CCGCTCGAGACCGATTTCCAGCTGAGAGTC
**MVN-BamHI For**	CGCGGATCCATGCCGAACTTCTCTCACACC
**MVN-BamHI Rev**	CGCGGATCCACCGATTTCCAGCTGAGAGTC
**MVN-EcoRI For**	CGCGAATTCATGCCGAACTTCTCTCACACC
**MVN-EcoRI Rev**	CGCGAATTCACCGATTTCCAGCTGAGAGTC
**MVN-SacI For**	CGCGAGCTCATGCCGAACTTCTCTCACACC
**MVN-SacI Rev**	CGCGAGCTCACCGATTTCCAGCTGAGAGTC
**MVN-BamHI6 For**	CGCGGATCC*GGCGGC*ATGCCGAACTTCTCTCACACC
**MVN-BamHI6 Rev**	CGCGGATCC*GGAGCC*ACCGATTTCCAGCTGAGAGTC
**MVN-EcoRI6 For**	CGCGAATTC*GGCGGC*ATGCCGAACTTCTCTCACACC
**MVN-EcoRI6 Rev**	CGCGAATTC*GGAGCC*ACCGATTTCCAGCTGAGAGTC
**MVN-SacI6 For**	CGCGAGCTC*GGCGGC*ATGCCGAACTTCTCTCACACC
**MVN-SacI6 Rev**	CGCGAGCTC*GGAGCC*ACCGATTTCCAGCTGAGAGTC
**MVN-BamHI10 For**	CGCGGATCC*GGCGGCTCCGGC*ATGCCGAACTTCTCTCACACC
**MVN-BamHI10 Rev**	CGCGGATCC*GCCGGAGCCGCC*ACCGATTTCCAGCTGAGAGTC
**MVN-EcoRI10 For**	CGCGAATTC*GGCGGCTCCGGC*ATGCCGAACTTCTCTCACACC
**MVN-EcoRI10 Rev**	CGCGAATTC*GCCGGAGCCGCC*ACCGATTTCCAGCTGAGAGTC
**MVN-SacI10 For**	CGCGAGCTC*GGCGGCTCCGGC*ATGCCGAACTTCTCTCACACC
**MVN-SacI10 Rev**	CGCGAGCTC*GCCGGAGCCGCC*ACCGATTTCCAGCTGAGAGTC
**RT-HCV Fw**	AGCCATGGCGTTAGTATGAGTGTC
**RT-HCV Rv**	ACAAGGCCTTTCGCAACCCAA
**RT-GAPDH Fw**	ACCACAGTCCATGCCATCAC
**RT-GAPDH Rv**	TCCACCACCCTGTTGCTGTA

Supplementary Table 2 Absorbance values of the elution fractions with the highest protein contents using Ni-NTA purification.

**MVN type**	**Monomer**	**2S**	**2M**	**2L**	**3S**	**3M**	**3L**	**4S**	**4M**	**4L**
UV Absorbance (280 nm)	0.393	0.460	0.390	0.424	0.426	0.387	0.209	0.198	0.158	0.550

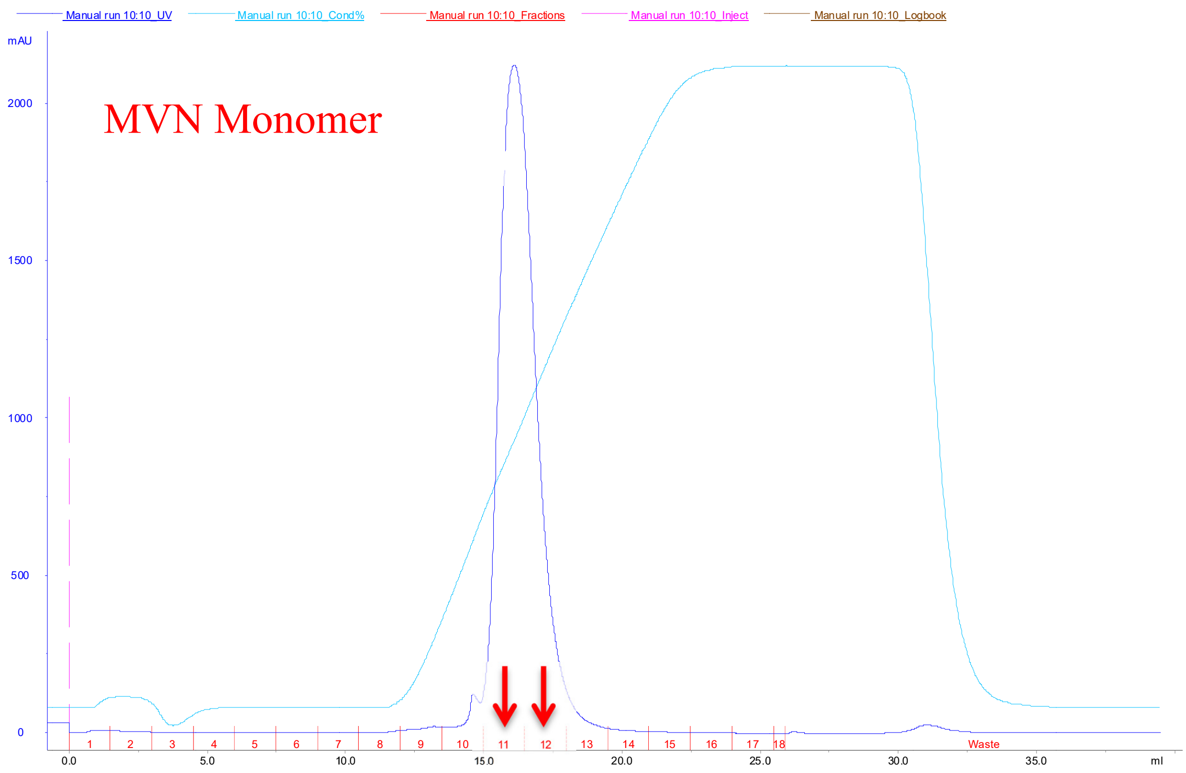

Supplementary Figure S1 FPLC chromatogram of the Q Sepharose XL ion exchange purification of the MVN monomer. Fractions 11 and 12 (red arrow) were collected and used for further experiments.

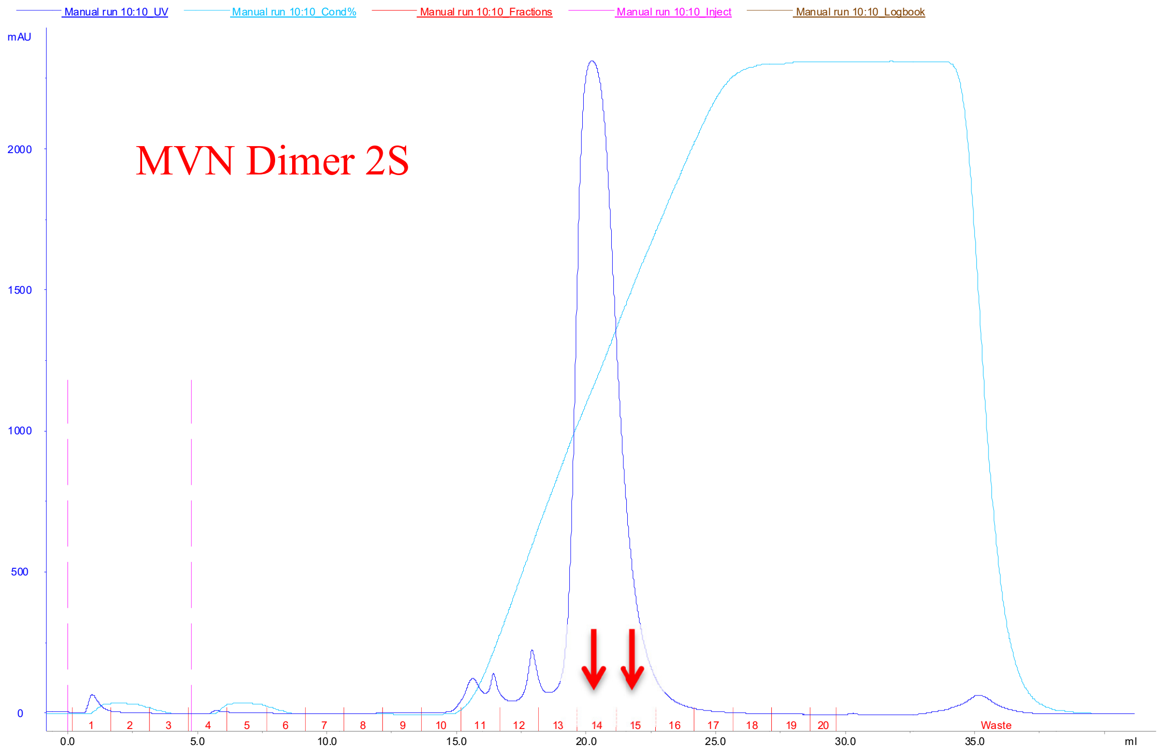

Supplementary Figure S2 FPLC chromatogram of the Q Sepharose XL ion exchange purification of the MVN dimer 2S. Fractions 14 and 15 (red arrow) were collected and used for further experiments.

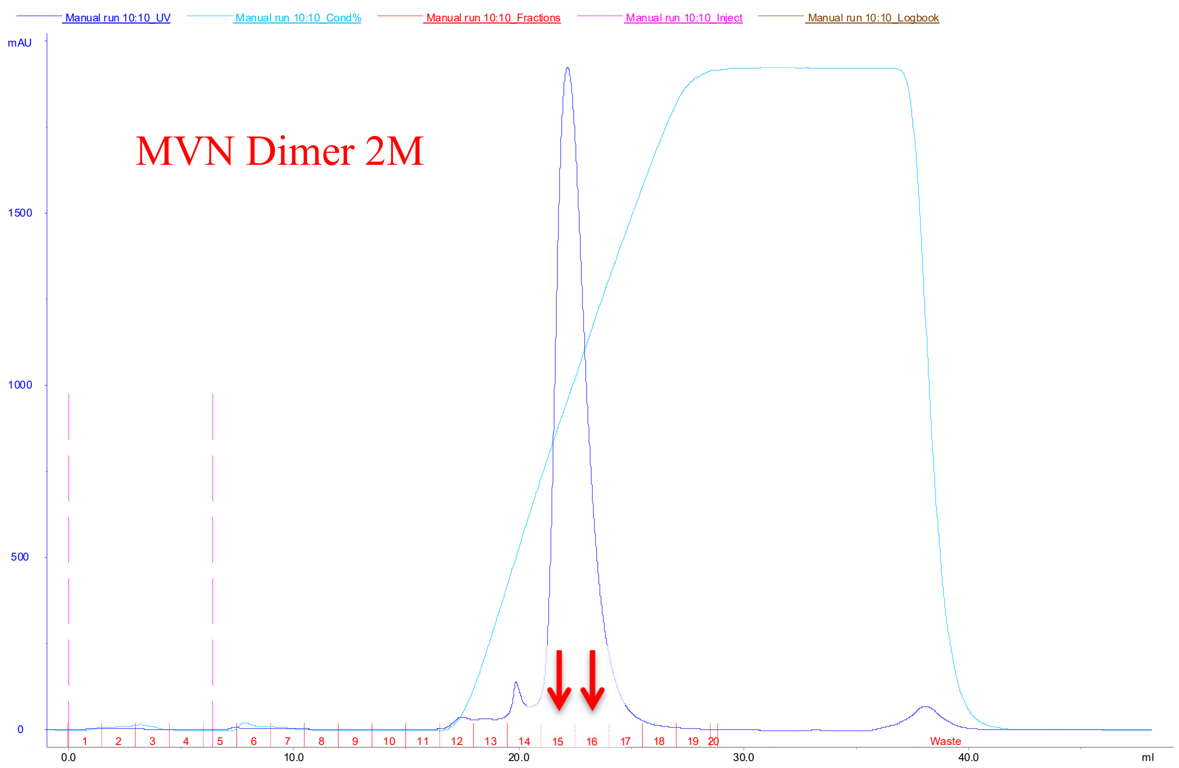

Supplementary Figure S3 FPLC chromatogram of the Q Sepharose XL ion exchange purification of the MVN dimer 2M. Fractions 15 and 16 (red arrow) were collected and used for further experiments.

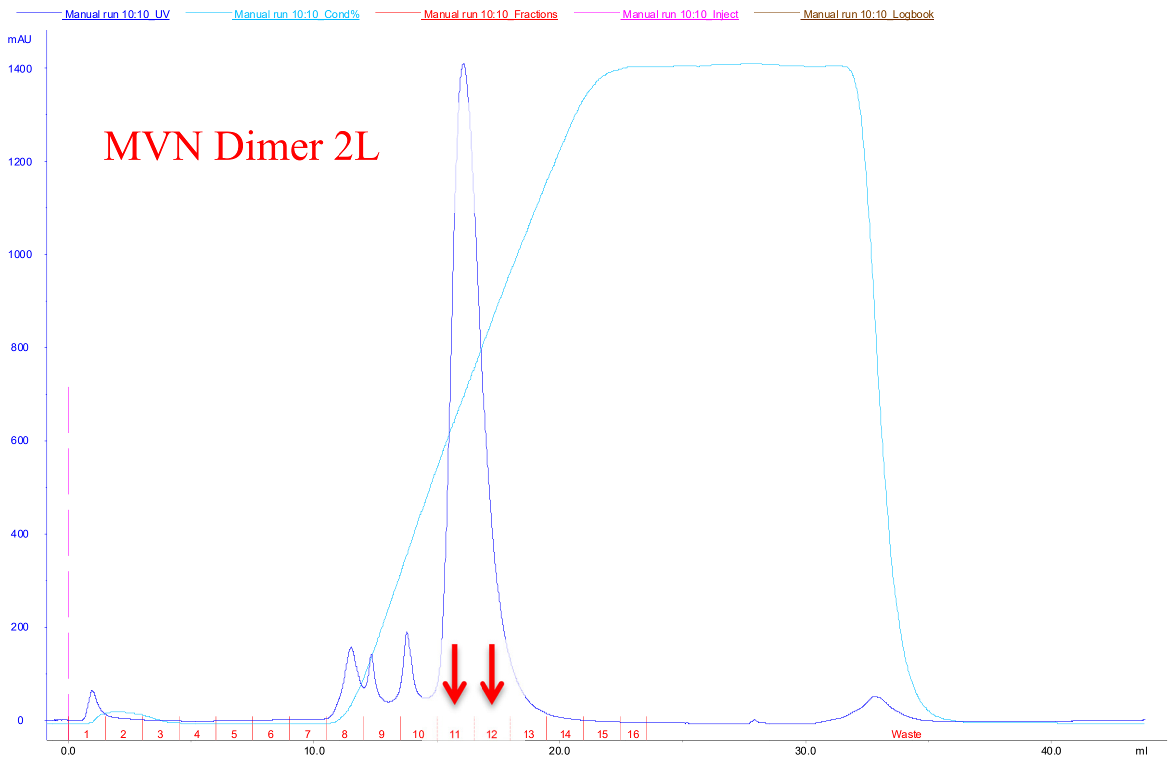

Supplementary Figure S4 FPLC chromatogram of the Q Sepharose XL ion exchange purification of the MVN dimer 2M. Fractions 11 and 12 (red arrow) were collected and used for further experiments.

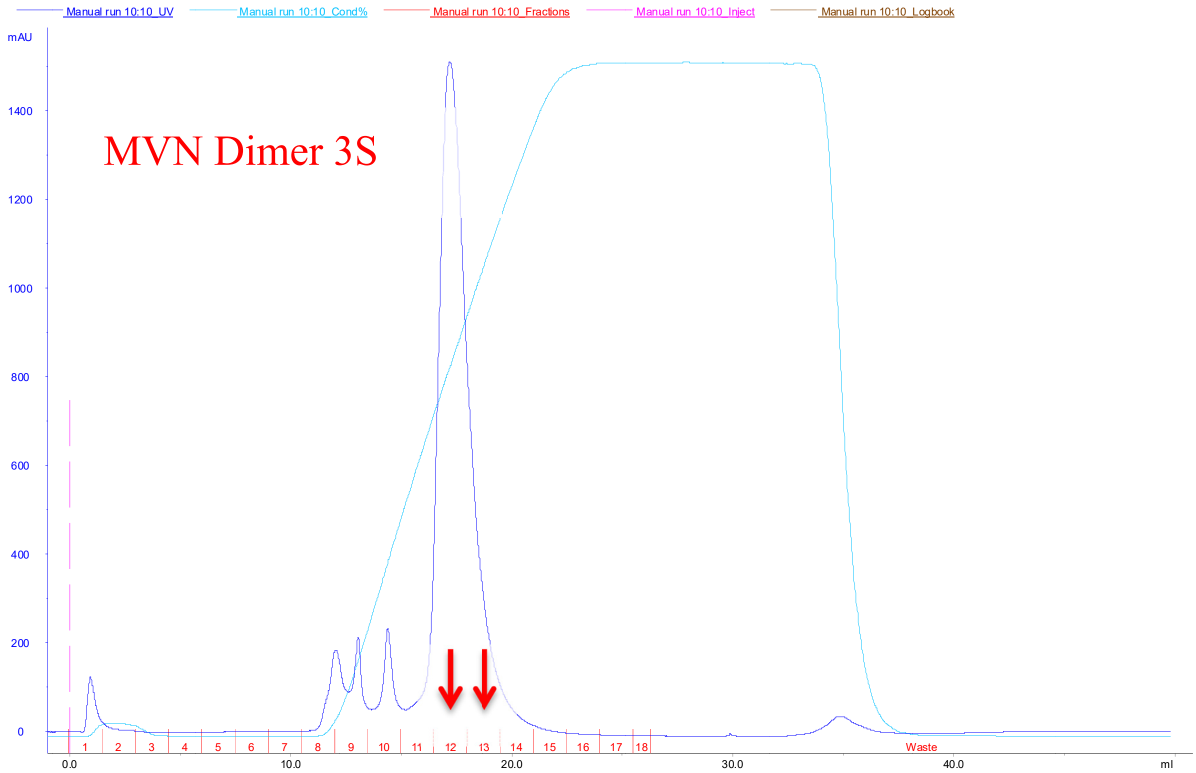

Supplementary Figure S5 FPLC chromatogram of the Q Sepharose XL ion exchange purification of the MVN trimer 3S. Fractions 12 and 13 (red arrow) were collected and used for further experiments.

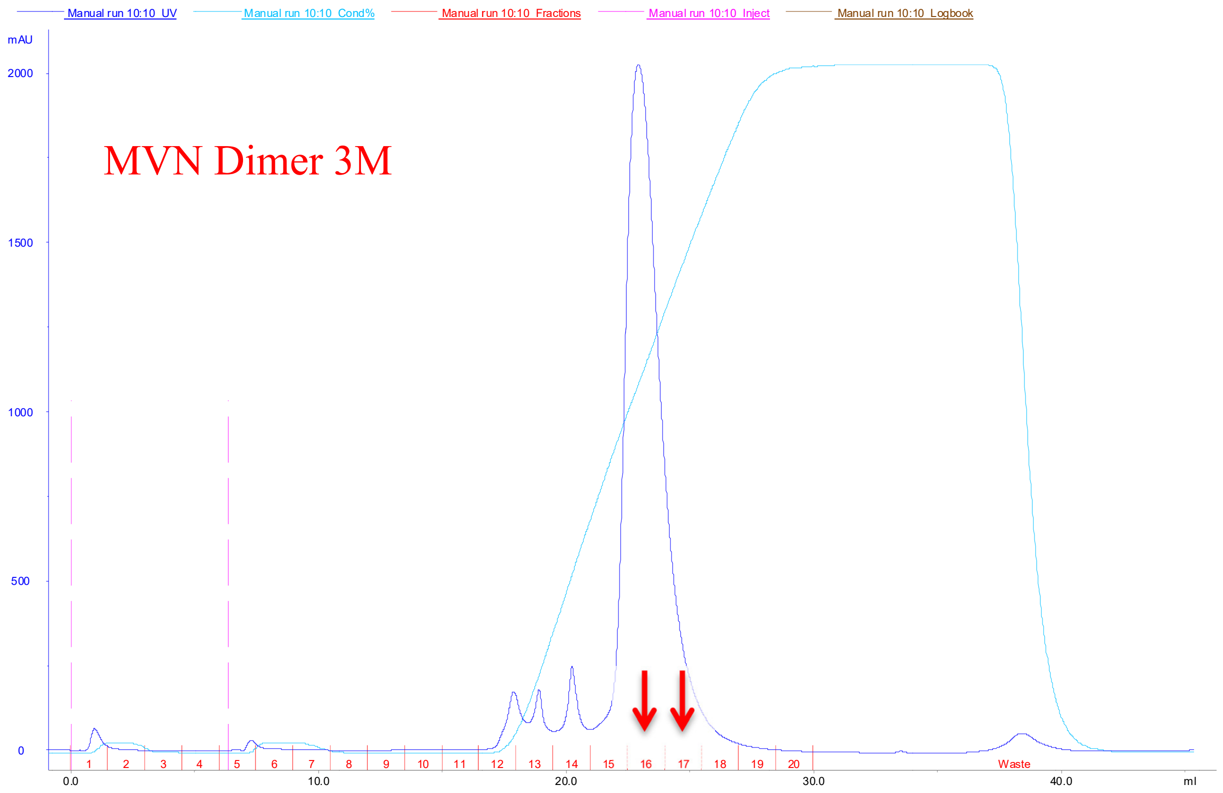

Supplementary Figure S6 FPLC chromatogram of the Q Sepharose XL ion exchange purification of the MVN trimer 3M. Fractions 16 and 17 (red arrow) were collected and used for further experiments.

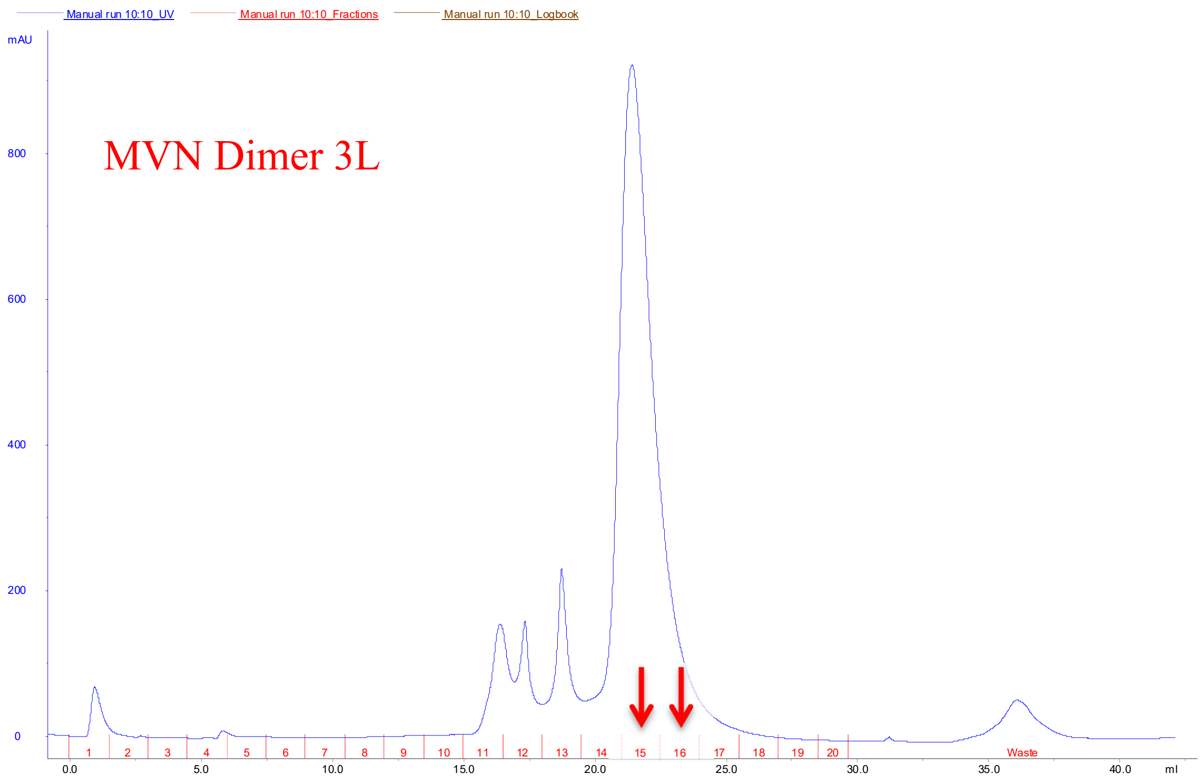

Supplementary Figure S7 FPLC chromatogram of the Q Sepharose XL ion exchange purification of the MVN trimer 3L. Fractions 15 and 16 (red arrow) were collected and used for further experiments.

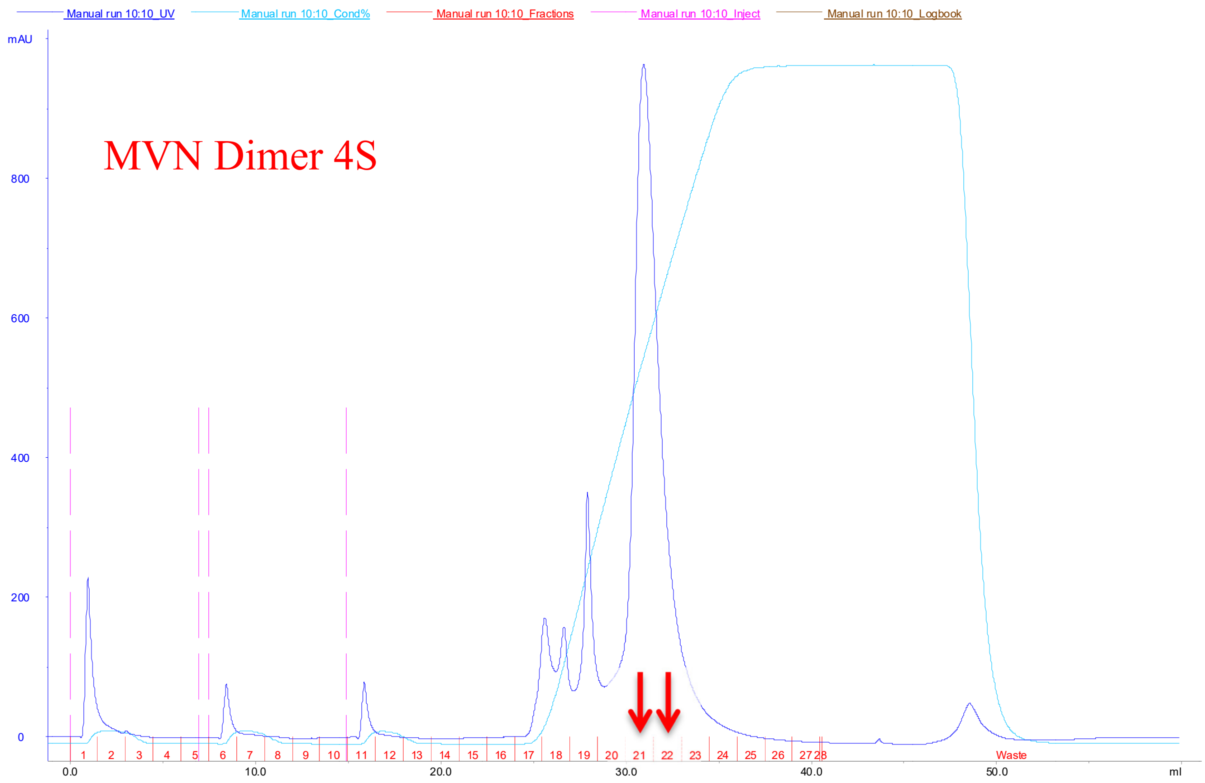

Supplementary Figure S8 FPLC chromatogram of the Q Sepharose XL ion exchange purification of the MVN tetramer 4S. Fractions 21 and 22 (red arrow) were collected and used for further experiments.

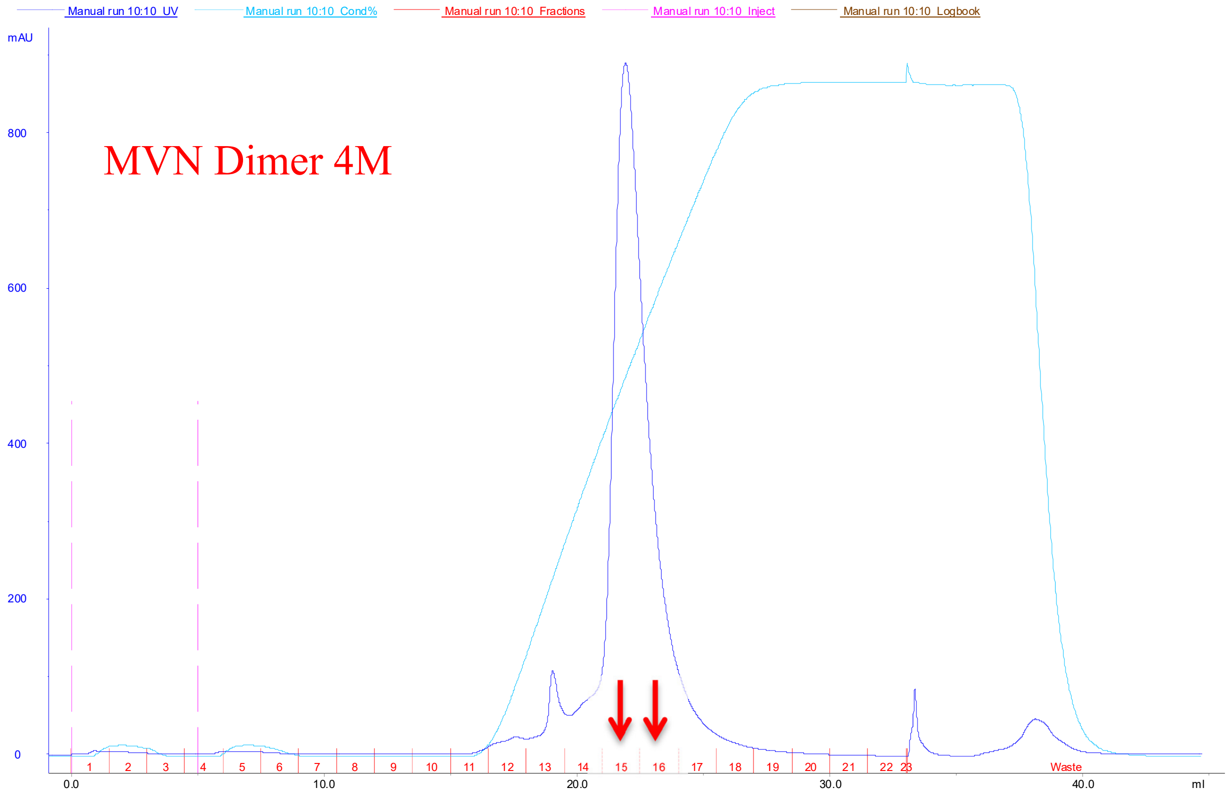

Supplementary Figure S9 FPLC chromatogram of the Q Sepharose XL ion exchange purification of the MVN tetramer 4M. Fractions 15 and 16 (red arrow) were collected and used for further experiments.

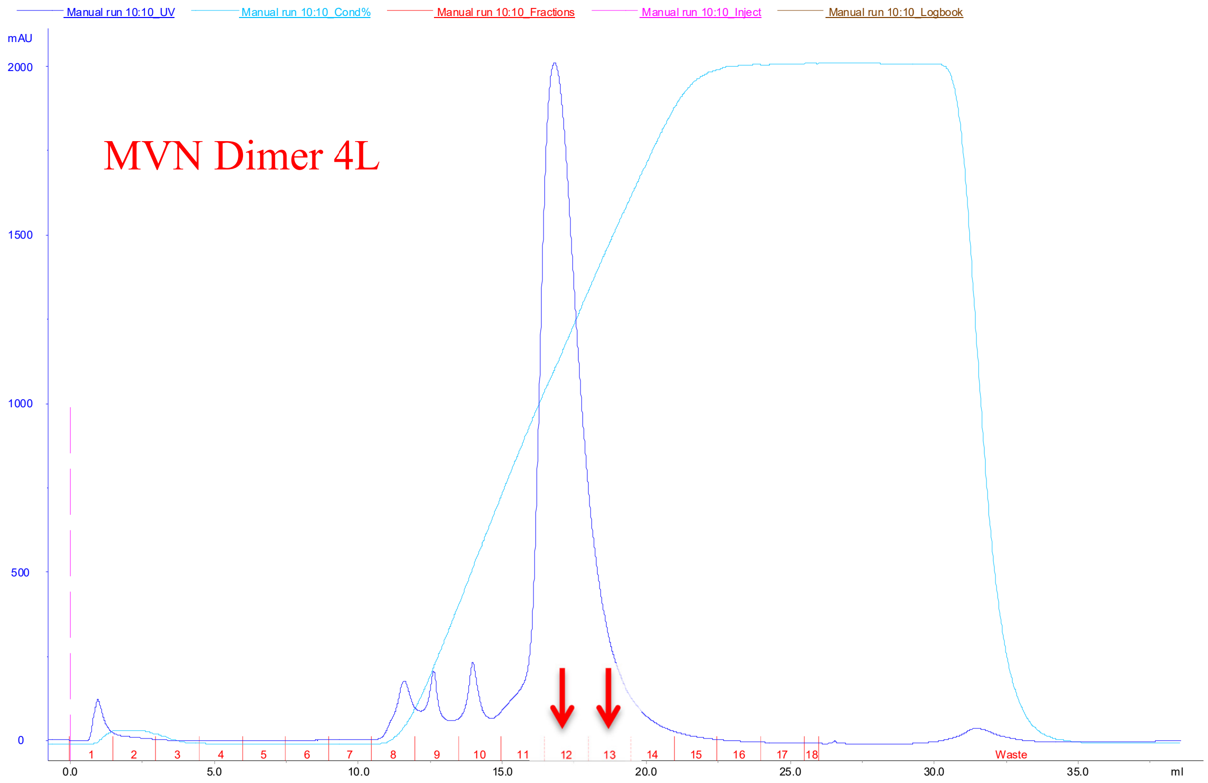

Supplementary Figure S10 FPLC chromatogram of the Q Sepharose XL ion exchange purification of the MVN tetramer 4L. Fractions 12 and 13 (red arrow) were collected and used for further experiments.

Supplementary Table 3 HCV N-glycan composition and possible isomers

**Retention time (min)**[a]	**GU**[a]	**GU (NIBRT)**[b]	**Hex**	**HexNAc**	**Fuc**	**m/z**[c]
**24.3**	6.11	6.18±0.02	5	2	-	1377.6
**27.6**	6.93	7.02±0.05	6	2	-	1539.5
**28.1**	7.08	7.20±0.02	5	4	-	1783.7
**29.5**	7.47	7.61±0.02	6	3	-	1742.7
**29.5**	7.47	7.61±0.02	5	4	1	1929.8
**31.9**	8.15	8.38±0.04	6	5	-	2148.8
**32.2**	8.28	8.33±0.00	6	4	1	2091.8

[a] Retention time and glucose unit (GU) values of 2-AB-labeled glycan species obtained by HILIC-UPLC

[b] The reference GU values from the National Institute for Bioprocessing Research and Training

[c] 2-AB-labeled glycan species ($[M+Na]^+$) analyzed by MALDI-TOF MS/MS

Hex= hexose; HexNAc=N-acetylhexosamine; Fuc=fucose

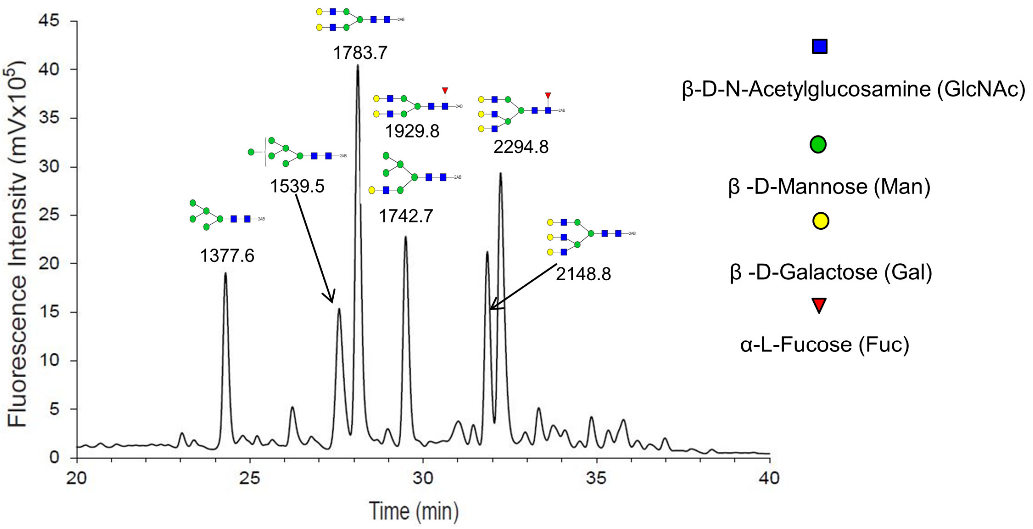

Supplementary Figure 11 UPLC chromatogram of HCV-derived N-glycans.

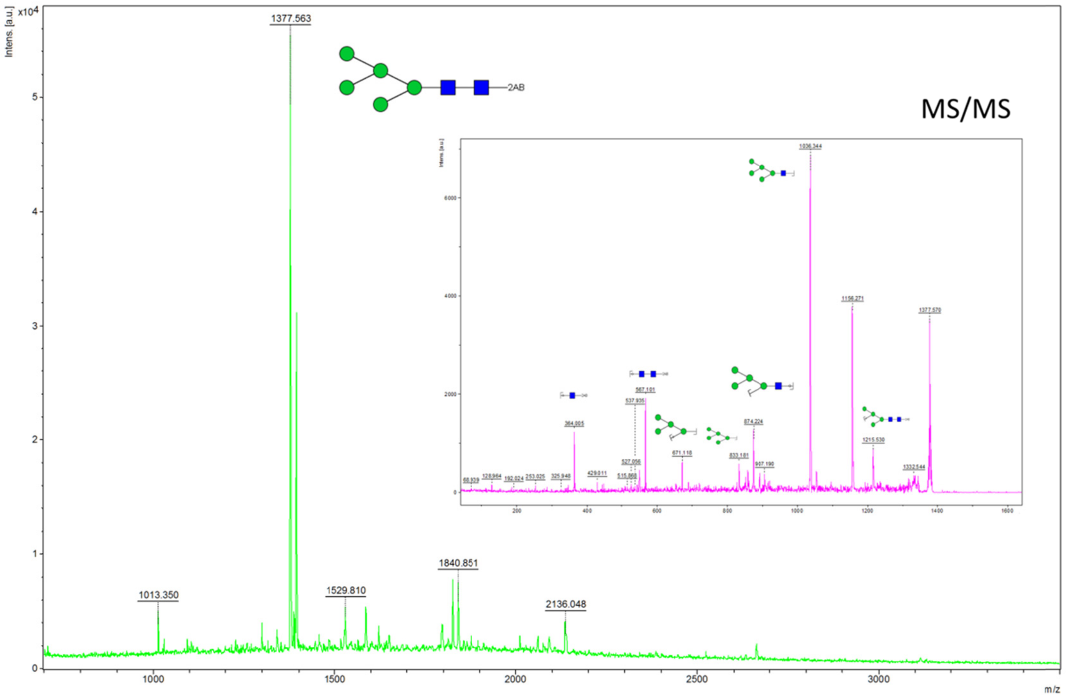

Supplementary Figure 12 Mass spectrometric analysis of UPLC elution peak at 24.3 min.

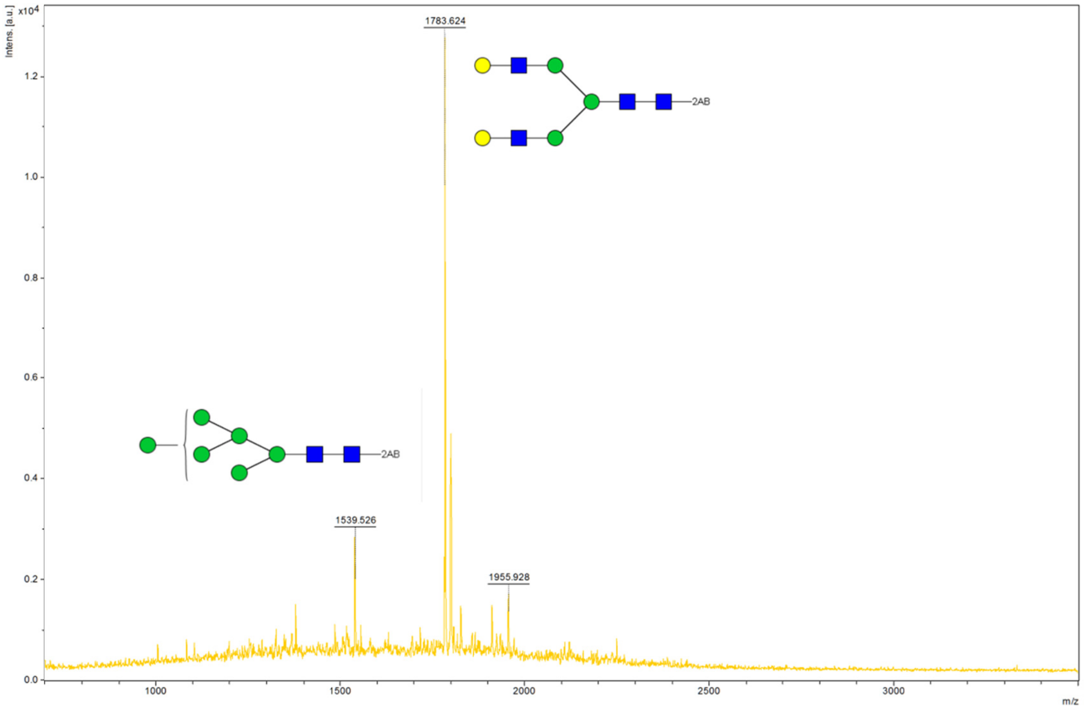

Supplementary Figure 13 Mass spectrometric analysis of UPLC elution peak at 27.6 min.

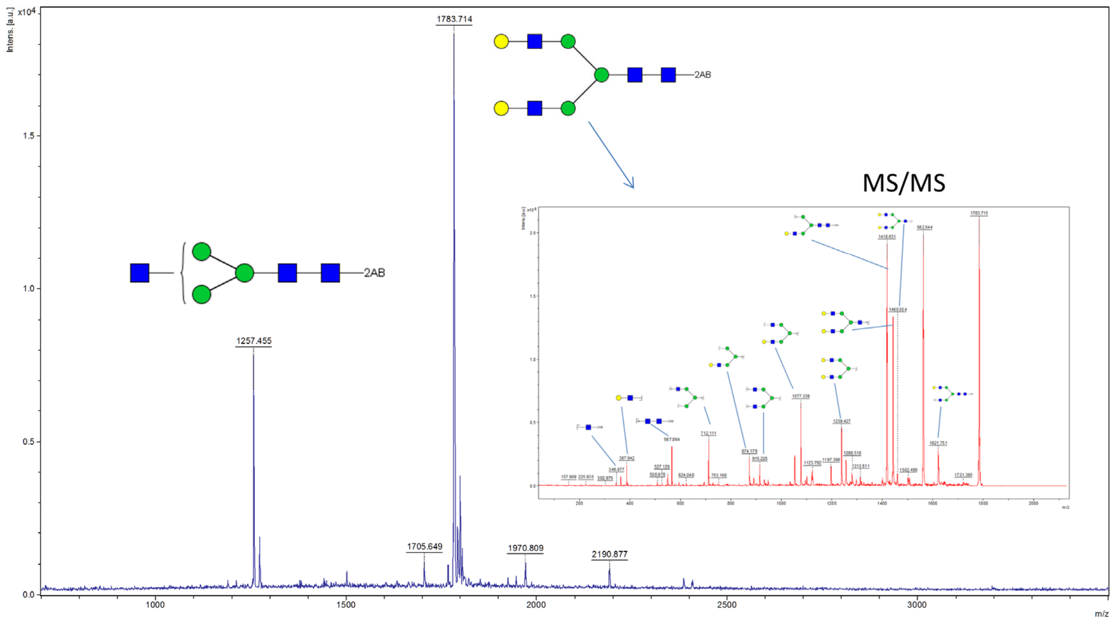

Supplementary Figure 14 Mass spectrometric analysis of UPLC elution peak at 28.1 min.

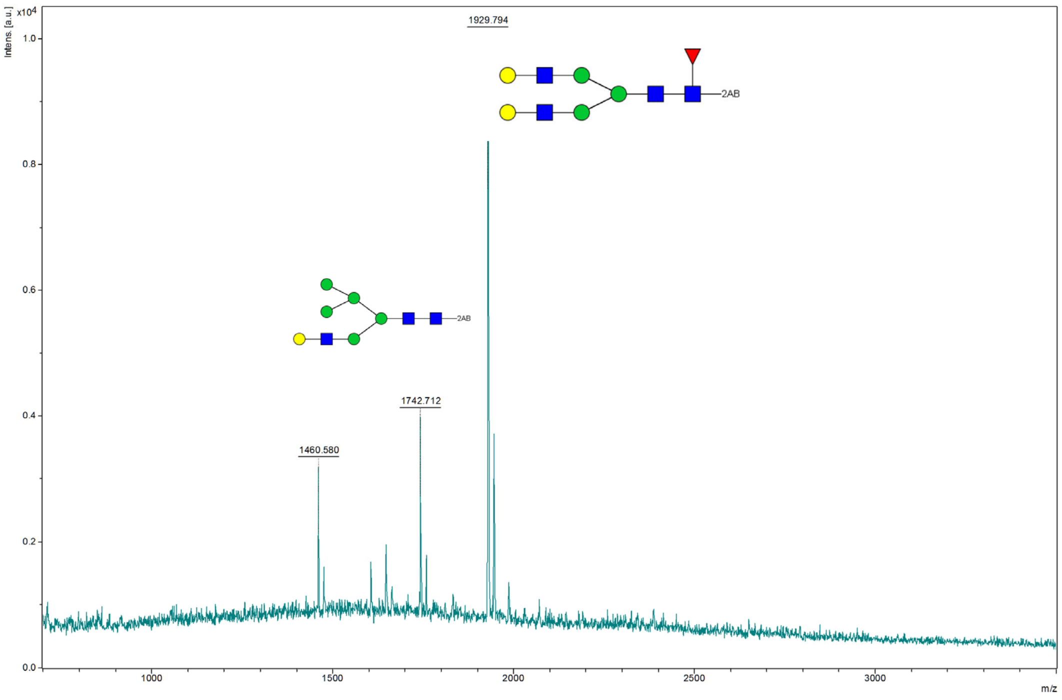

Supplementary Figure 15 Mass spectrometric analysis of UPLC elution peak at 29.5 min.

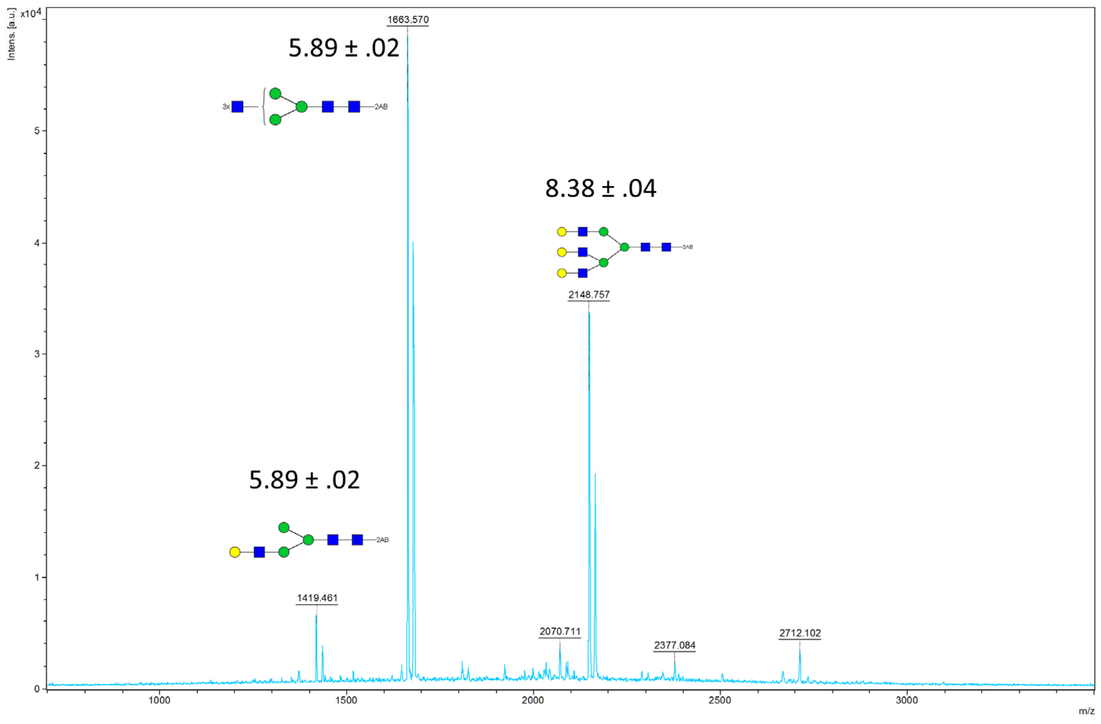

Supplementary Figure 16 Mass spectrometric analysis of UPLC elution peak at 31.9 min.

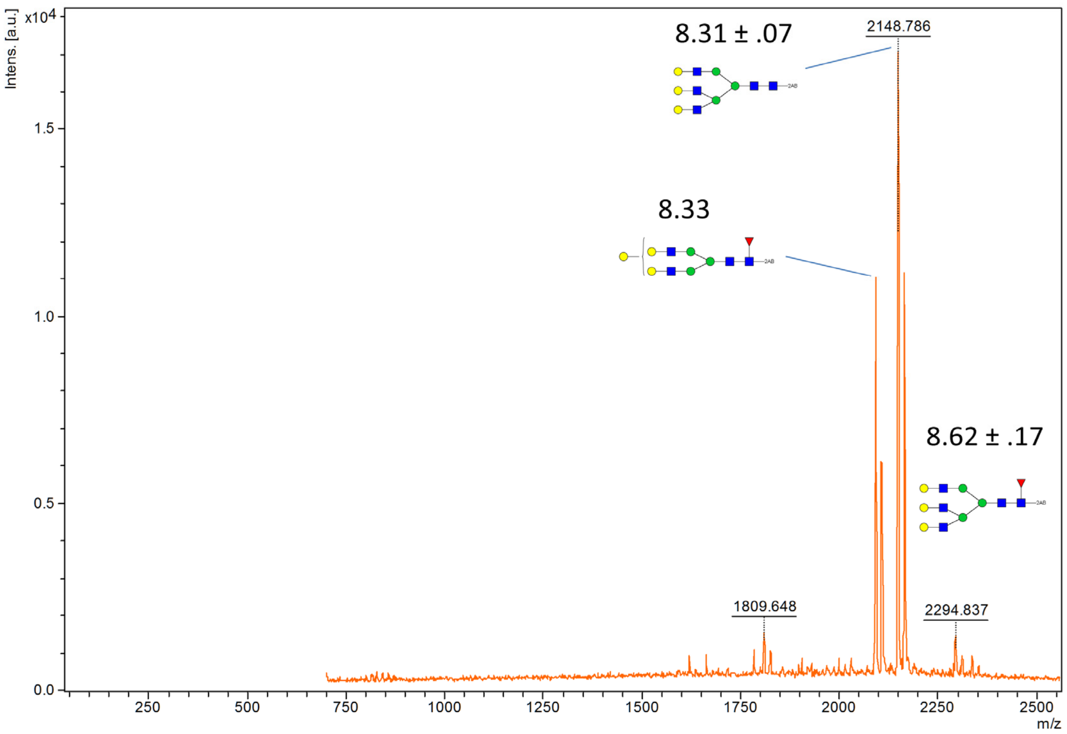

Supplementary Figure 17 Mass spectrometric analysis of UPLC elution peak at 32.2 min.
